# Change in Surface Roughness of Esthetic Restorative Materials after Exposure to Different Immersion Regimes in a Cola Drink

**DOI:** 10.1155/2014/353926

**Published:** 2014-03-23

**Authors:** Navroop Kaur Bajwa, Anuradha Pathak

**Affiliations:** ^1^Pedodontics and Preventive Dentistry, Punjab Civil Medical Services (Dental), Government of Punjab, House No. 1662, Sector 70, Mohali, Punjab 160071, India; ^2^Department of Pedodontics and Preventive Dentistry, Government Dental College and Hospital, House No. 5094, Phase 1, Urban Estate, Patiala, Punjab 147002, India

## Abstract

*Context*. An in vitro study carried out to evaluate and compare the effect of Cola drink on surface roughness of esthetic restorative materials. *Purpose*. To compare the effect of different immersion regimes in a Cola drink on surface roughness of esthetic restorative materials. *Method*. Two hundred samples were grouped into 4 equal groups of 50 samples each: *Group I*: conventional glass ionomer, *Group II*: resin modified glass ionomer, *Group III*: polyacid-modified resin composite, *Group IV*: Composite resin. Each group was further subdivided into 5 subgroups of 10 samples each. *Subgroup A (Control Subgroup)*. Samples were kept immersed in artificial saliva. *Subgroup B*. Samples were immersed in Cola drink once a day. *Subgroup C*. Samples were immersed in Cola drink, 3 times a day. *Subgroup D*. Samples were immersed in Cola drink 5 times a day. *Subgroup E*. Samples were immersed in Cola drink 10 times a day. Each immersion lasted 5 minutes. The immersion protocol was repeated for 7 days. *Results*. Maximum surface roughness was seen in Group I conventional glass ionomer cement, followed by Group II resin modified glass ionomer, Group III polyacid modified resin composite, and Group IV composite resin samples. *Conclusion*. Resistance to change in surface roughness is more in resin based restorative materials as compared to glass ionomer based materials.

## 1. Introduction

The mouth is considered as the ideal environment for predicting the behavior of restorative materials [1]. Restorative filling materials used in dentistry are required to have long-term durability in the oral cavity [2]. Considering that tooth colored restorative materials are commonly used for restorations in children and adolescents, who in turn are major consumers of soft drinks, it is important not only to compare the performance of different restorative materials but also to estimate their chemical durability. Under acidic conditions, restorative materials suffer degradation over time, which can be predicted by changes in the surface roughness. Studies reporting the association of frequency of soft drink ingestion have shown an increased degradative potential with the increase in frequency of consumption [3]. Due to the complexity and diversity of intraoral conditions, in vitro models are very important for providing an insight into the fundamental mechanisms of biodegradation [1].

Therefore this in vitro study was conducted to evaluate and compare the effect of Cola drink (Coca-Cola), on the surface roughness of tooth colored restorative materials after exposure to different immersion regimes.

## 2. Materials and Method

The study investigated the effect of different immersion regimes in a Cola drink on the surface roughness (*R*_*a*_, *μ*m) of a conventional glass ionomer, resin modified glass ionomer, a polyacid modified resin composite, and a nanofilled composite resin (Table 1). Fifty samples were prepared from each restorative material resulting in a total of 200 samples using a ring shaped brass mould of diameter 10 mm × 2 mm height. This ensured the standardization of shape and size of each sample. Samples were made by introducing sufficient amount of material into the mould and pressing between mylar matrix strips supported by glass slides on either side. Only a polyester strip and a glass slide were used while making the samples before lights polymerization or setting with the intention of obtaining a smooth and flat surface. Any form of additional polishing can lead to an increase in surface roughness and hence no polishing of the samples was carried out. Samples were grouped into 4 equal groups of 50 samples each.

*Group I.* Conventional glass ionomer cement (GC Fuji II).

*Group II.* Resin modified glass ionomer cement (GC Fuji II LC).

*Group III.* Polyacid-modified resin composite (Dyract Extra). 

*Group IV.* Composite resin (Filtek Z 350). 

Samples in each group were further divided into five subgroups of 10 samples each (Table 2). 

*Subgroup A (Control Subgroup).* Each sample was kept completely immersed in 25 mL of artificial saliva in an air tight container at room temperature for 7 days. Artificial saliva in each container was changed every day.

Samples in the remaining 4 subgroups were immersed in Cola drink (Coca-Cola) and subjected to four different immersion regimes. On an average, a child can be assumed to consume soft drinks once or thrice during the time he is awake in a day. Towards the higher end of the continuum, soft drink consumption may be 5 times or 10 times per 12 hours. Hence, in this study the above frequencies of consumption were used as the yardsticks for deciding the number of immersions per day in vitro. Waking period in children is assumed to be approximately 12 hours in a day. Hence, the immersions were evenly distributed over a 12 hour period. Before and after immersion in the Cola drink (Coca-Cola), samples in these subgroups were copiously rinsed in 0.1 M phosphate buffered saline (PBS) pH = 7.2 [3].

This was done for the following reasons:

- to buffer the effect of Cola drink (Coca-Cola) after the prescribed exposure time;
- to return the pH to a neutral level once the exposure was over;
- to avoid prolonged insult to the materials while they were stored in the artificial saliva.

*Subgroup B*. Samples were immersed in 25 mL of Cola drink once a day. 

*Subgroup C.* Samples were immersed in 25 mL of Cola drink, 3 times a day. 

*Subgroup D.* Samples were immersed in 25 mL of cola drink 5 times a day.

*Subgroup E.* Samples were immersed in 25 mL of Cola drink 10 times a day. 

In this study, the samples were completely immersed in Cola drink (Coca-Cola) for 5 minutes in contrast to in vitro studies that had employed extremely long time intervals of immersion in eroding solutions ranging from 15 to 180 minutes [4]. The container was agitated continuously for 5 minutes during the immersion to ensure complete contact of the samples with the immersion medium (Coca-Cola) and also to simulate intraoral conditions because at least some degree of agitation occurs intraorally as well while drinking. Cola drink used for immersion of samples was changed after each immersion. The samples in subgroups B, C, D, and E of all the groups were kept immersed in artificial saliva in air tight containers when not subjected to the immersion regime. Although water has been a commonly employed storage medium in in vitro studies, this study preferred artificial saliva over distilled water as the storage medium to simulate the oral environment and provide data closer to reality and reproduce clinical situations. Composition of artificial saliva as proposed by Klimek et al. [5] was used: 0.002 g ascorbic acid, 0.030 g glucose, 0.580 g NaCl, 0.170 g CaCl_2_, 0.160 g NH_4_Cl, 1.270 g KCl, 0.160 g NaSCN, 0.330 g KH_2_PO_4_, 0.200 g urea, 0.340 g Na_2_HPO_4_, 2.700 g mucin in 1000 mL distilled water. The solution was further titrated with a phosphate buffer of 26.4 mL 0.06 M Na_2_HPO_4_·2H_2_O and 7.36 mL 0.06 M KH_2_PO_4_.

As the greatest change in physical properties has been shown to occur within the first 7 days of exposure to solutions; hence, the immersion protocol was carried on for 7 days [4].

Each sample was blot dried and subjected to surface roughness testing by a Profilometer and the value (*R*_*a*_) was obtained in *μ*m.

The data obtained was compiled and put to statistical analysis. One way ANOVA test was used as the study had more than 2 groups and the data obtained was normally distributed. For intergroup comparison, multiple comparison post-hoc test (Turkey HSD) was employed.

## 3. Results

**Figure 1:**
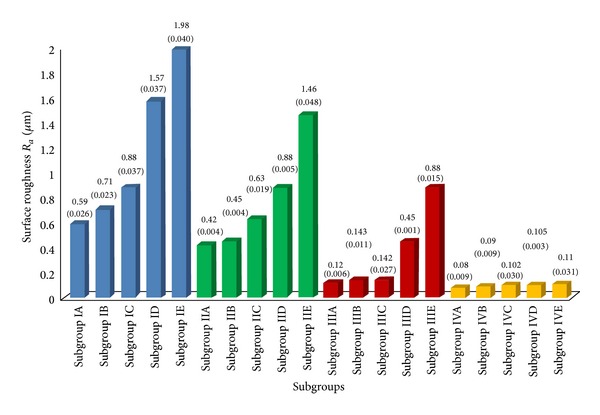
Comparison of the effect of different Cola drink immersion regimes on surface roughness (*R*_*a*_, *μ*m) of restorative materials tested (mean values) and standard deviation (SD).

The mean and standard deviation were calculated for the comparison of surface roughness of all subgroups of groups I, II, III, and IV (Figure 1). Maximum surface roughness was seen in Group I conventional glass ionomer cement (GC Fuji II), followed by Group II resin modified glass ionomer cement (GC Fuji II LC), and Group III polyacid modified resin composite (Dyract Extra) and least surface roughness was seen in Group IV composite resin samples (Filtek Z 350).

Resistance to change in surface roughness was seen in the following sequence: Group IV Composite resin (Filtek Z 350) > Group III Polyacid modified resin composite (Dyract Extra) > Group II Resin modified glass ionomer cement (GC Fuji II LC) > Group I conventional glass ionomer cement (GC Fuji II). As the number of immersions increased, each consecutive subgroup showed highly significant increase in surface roughness.

In* Group I (conventional glass ionomer cement-GC Fuji II),* highly significant increase in surface roughness values (*P* < 0.01) was observed in subgroups IB (1 immersion/day), IC (3 immersions/day), ID (5 immersions/day), and IE (10 immersions/day) on comparison with subgroup IA (control) (Table 3). In* Group II (resin modified glass ionomer cement-GC Fuji II LC)*, subgroups IIC (3 immersions/day), IID (5 immersions/day), and IIE (10 immersions/day) showed highly significant increase in surface roughness values (*P* < 0.01) while subgroup IIB (1 immersion/day) showed insignificant increase (*P* > 0.05) on comparison with subgroup IIA (control) (Table 4). In case of* Group III (polyacid modified resin composite-Dyract Extra)* subgroups IIID (5 immersions/day) and IIIE (10 immersions/day) showed highly significant increase in surface roughness values (*P* < 0.01) while subgroup IIIB (1 immersion/day) and IIIC (3 immersions/day) showed insignificant increase (*P* > 0.05) on comparison with subgroup IIIA (control) (Table 5). In case of* Group IV composite resin (Filtek Z 350)* subgroups IVB (1 immersion/day), IVC (3 immersions/day), IVD (5 immersions/day), and IVE (10 immersions/day) showed insignificant increase in surface roughness values (*P* > 0.05) on comparison with IVA (control) (Table 6).

## 4. Discussion

Roughening can be a consequence of the chemical dissolution of restorative materials by exposure to chemicals from acidic drinks and acidic food items. According to Hamouda [6], the roughness of all intra-oral hard surfaces should approximate a *R*_*a*_ value of 0.2 *μ*m or lower to reduce bacterial retention. Residual surface roughness of restorations encourages plaque accumulation. *R*_*a*_ value (surface roughness) is the arithmetic mean of the departures of the roughness profile from the mean line [6].

It has been established that the erosive potential of an acidic solution is related to its pH, titratable acidity and buffer capacity. pH of Coca-Cola is low that is, ~2.5. In addition, this soft drink has in its composition an inorganic and strong acid, phosphoric acid. Thus, the association of a low pH and the presence of a strong inorganic acid could have caused a more aggressive attack on the surface of restorative materials hence leading to an increase in the surface roughness.

In the control subgroups, maximum surface roughness was seen in conventional glass ionomer cement followed by resin modified glass ionomer cement, polyacid modified resin composite and least surface roughness was seen in composite resin samples. This finding is supported by Gladys et al. [7] who showed that in their study, composite resins showed the smoothest surface followed by compomer (5–20 times rougher than composites), resin modified glass ionomers and glass ionomers (14–25 times higher roughness than composites) showing the maximum surface roughness.

The results showed that the surface roughness values of all the restorative materials immersed in the Cola drink (Coca-Cola) increased as the number of immersions increased. Greater number of immersions in the Cola drink (Coca-Cola) resulted in a more accentuated impact on the restorative materials. Also the resistance to change in surface roughness was seen in the following sequence.

Composite resin (Filtek Z 350) > Polyacid modified resin composite (Dyract Extra) > Resin modified glass ionomer cement (GC Fuji II LC) > Conventional glass ionomer cement (GC Fuji II).

These results are in concurrence with those of Hamouda [6], who concluded that the conventional glass ionomer showed the highest surface roughness in the acidic media followed by the resin modified glass ionomer and compomer restorative materials.

These findings also coincide with the in vitro study conducted by EI-Korashyand and Mobarak [8].

These findings are supported by Briso et al. [1] who conducted a study to evaluate the effect of different acidic solutions on the microhardness and surface roughness of resin modified glass ionomer and composite resin (sealed with surface sealant and unsealed). Results showed that resin modified glass ionomer cement showed the highest change in surface roughness values after immersion in acidic media, whereas the lowest values were found for the composite resin before and after exposure.

Glass ionomer based materials showed more surface roughness in all subgroups in comparison to composite resin based materials. It can be explained by the fact that conventional glass ionomer cement consists of glass particles in a hydrogel matrix. In acidic solutions, H^+^ ions of the acid diffuse into the bulk of the restoration and replace metal cations in the matrix. These free cations then diffuse outward and are released from the surface. As the metal cations in the matrix decrease, more ions are extracted from the surrounding glass particles, causing them to dissolve. Consequently the material presents a rough surface with voids and protruded, undissolved glass particles. H^+^ ion concentration and the formation constants for soluble complexes between acid anions and metal cations in the set cement control the degree of glass-ionomer cement's erosion in organic acid solutions.

Comparing the hybrid restorative materials, resin modified glass ionomer shows higher surface roughness values than compomer. This is related to the higher filler particle size in resin modified glass ionomers. Also, unlike polyacid-modified resin composites, the coherence between the cross-linked polyacrylate network and the polymer chain of resin-modified glass ionomers seems insufficient. When exposed to low pH in an aqueous environment, resin modified glass ionomers may take up a lot of water, swell and become plastic and mechanically less resistant to surface degradation than other resin-based materials [9].

These results are supported by an in-vitro study conducted by Mohamad-Tahir and Yap [10] who concluded that at various acidic pH (2, 3, 4, 5, and 6), polyacid modified resin composite showed minimal changes in surface roughness values. These findings can be explained as follows.

Polyacid modified resin composite is an anhydride, which could react with water of the storage medium, showing the development of a carboxylate rich surface on the uppermost layer, rendering this material more resistant to degradation than conventional and resin modified glass ionomers.

Nonetheless, statistically significant increase in surface roughness observed on exposure of Dyract to high frequency of immersion could be due to hydrolysis of the silane-coupling agent or the plasticizing process of the resin matrix caused by prolonged immersion in acidic medium. The poorer silanization of the filler particles can also increase the potential for filler particle debonding.

Filtek Z 350 samples showed the minimum surface roughness values in all subgroups. These findings corroborated with those of Gladys et al. [7] and Turssi et al. [11].

Composites with small filler particles are more homogeneous and their particles are less prominent on the surface, resulting in a lower surface roughness. Whereas the type of filler and size and quantity of the particles influence the properties and quality of polishing of composite resins, the reduction in space between the inorganic nano-clusters is possibly responsible for superior physical properties of nano-filled composites (Filtek Z 350). This result also agrees with the results of Soderholm [12] and Han et al. [13], who suggested that relatively higher filler loading increases the stability of resin composite surface against low pH conditions.

However, still an insignificant increase in surface roughness of Filtek Z 350 samples.

These changes may be due to sorption of water by composite resin Filtek Z350 under acidic conditions leading to an increase in roughness, as it is composed predominantly of monomers that are more susceptible to hydrolysis, that is, Bis-GMA and TEGDMA. But the particles that are stripped out from the surface are very small; hence, they leave small holes which produce an insignificant increase in roughness. This finding coincides with that of Bagheri et al. [14] and Valinoti et al. [15].

## 5. Conclusion

The results of the present study tend to support the suggestion that the detrimental effect of Cola drink (Coca-Cola) on the surface properties of restorative materials is related to the frequency of consumption. Also, in clinical decision-making, a material with less resistance to degradative effects of acidic beverages may not be suitable for patients who have the habit of frequent consumption of soft drinks. Nevertheless, this research was conducted in vitro, whereas the effect of Cola drink on restorative materials may be modified by variables that are reproduced in vivo. To take into account the dynamic factors present in the oral cavity, further studies combining both qualitative and quantitative evaluations are necessary which will indicate more precisely the effects of acidic beverages on the clinical integrity of the restorative materials.

## Figures and Tables

**Table 1:** Description of the restorative materials used in the study.

Material	Manufacturer	Category	Composition	Shade used
FUJI II	GC Corporation, Japan	Conventional glass ionomer cement	Powder: calcium aluminum ESPE Premier fluorosilicate glass, pigments Liquid: polycarbonic acid, tartaric acid, water, benzoic acid (as a preservative)	A2

FUJI II LC	GC Corporation, Japan	Resin modified glass ionomer cement	Distilled water, polyacrylic acid, 2-hydroxyethylmetacrylate, urethane dimethacrylate, camphorquinone, fluoroaluminosilicate filler	A2

DYRACT EXTRA	Dentsply DeTrey GmbH, Konstanz, Germany	Polyacid modified resin composite	Urethane dimethacrylate, carboxylic acid modified dimethacrylate, triethylene glycol dimethacrylate, trimethacrylate resin, highly dispersed silicon dioxide, strontium-alumino-sodiumfluoro-phosphosilicate glass, strontium fluoride	A2

FILTEK Z 350	3M ESPE	Nanofilled composite resin	Organic matrix (% w) Bis-GMA (10–15), UDMA, TEGDMA (10–15) eBis-EMA (1–5) Inorganic filler (% w.v − 1) Nanoagglomerate of zirconia/silica (0.6 *μ*m a 1.4 *μ*m); Silica not agglomerated/not aggregated (20 nm) (78.5/59.5)	A2

**Table 2:** Shows the division of the samples into respective groups and further subgroups according to the immersion protocol.

Groups	Subgroup A (control)	Subgroup B 1 immersion/day	Subgroup C 3 immersions/day	Subgroup D 5 immersions/day	Subgroup E 10 immersions/day
I	10 samples	10 samples	10 samples	10 samples	10 samples
II	10 samples	10 samples	10 samples	10 samples	10 samples
III	10 samples	10 samples	10 samples	10 samples	10 samples
IV	10 samples	10 samples	10 samples	10 samples	10 samples

**Table 3:** Showing mean difference in surface roughness (*R*_*a*_, *μ*m) among various subgroups of group I—conventional glass ionomer cement (GC Fuji II).

Subgroup	Subgroup	Surface roughness (*R*_*a*_, *μ*m)		
		Mean difference	(*P* value)	Significance
Subgroup IA (Control)	Subgroup IB	−0.11	<0.001	HS
	Subgroup IC	−0.28	<0.001	HS
	Subgroup ID	−0.97	<0.001	HS
	Subgroup IE	−1.38	<0.001	HS

**Table 4:** Showing mean difference in surface roughness (*R*_*a*_, *μ*m) among various subgroups of group II—resin modified glass ionomer cement (GC Fuji II LC).

Subgroup	Subgroup	Surface roughness (*R*_*a*_, *μ*m)		
		Mean difference	(*P* value)	Significance
Subgroup IIA (Control)	Subgroup IIB	−0.02	>0.05	NS
	Subgroup IIC	−0.21	<0.001	HS
	Subgroup IID	−0.45	<0.001	HS
	Subgroup IIE	−1.04	<0.001	HS

**Table 5:** Showing mean difference in surface roughness (*R*_*a*_, *μ*m) among various subgroups of group III—DYRACT extra.

Subgroup	Subgroup	Surface roughness (*R*_*a*_, *μ*m)		
		Mean difference	(*P* value)	Significance
Subgroup IIIA (Control)	Subgroup IIIB	−0.018	>0.05	NS
	Subgroup IIIC	−0.017	>0.05	NS
	Subgroup IIID	−0.33	<0.001	HS
	Sub group IIIE	−0.76	<0.001	HS

**Table 6:** Showing mean difference in surface roughness (*R*_*a*_, *μ*m) and microhardness (HV) among various sub groups of group IV—composite resin (Filtek Z 350).

Subgroup	Subgroup	Surface roughness (*R*_*a*_, *μ*m)		
		Mean difference	(*P* value)	Significance
Subgroup IVA (Control)	Subgroup IVB	−0.014	>0.05	NS
	Subgroup IVC	−0.018	>0.05	NS
	Subgroup IVD	−0.022	>0.05	NS
	Subgroup IVE	−0.029	>0.05	NS
